# Unusual Pancreatic Abscess Secondary to Embedded Fish Bone: A Challenging Clinical Scenario

**DOI:** 10.3390/diagnostics12122999

**Published:** 2022-11-30

**Authors:** Yu-Jie Wu, Ying-Ying Chen, Yi-Chien Hsieh

**Affiliations:** 1 Department of Radiology, Shuang Ho Hospital, Taipei Medical University, New Taipei City 235041, Taiwan; 2 Department of Radiology, Wan Fang Hospital, Taipei Medical University, Taipei 116079, Taiwan

**Keywords:** fish bone, foreign body, pancreas, laparoscopic surgery

## Abstract

The incidental ingestion of fish bone is common, and the ingested fish bone mostly exits the gastrointestinal tract spontaneously. However, severe complications such as perforation in the digestive tract and abscess formation after a period of time may occasionally occur. Fewer than 10 cases of a migrated fish bone penetrating into the pancreas have been reported in the literature, and the development of a subsequent pancreatic abscess is extremely rare. We present one such rare case of pancreatic abscess formation in a middle-aged woman due to fish bone penetration through the gastric wall into the pancreas 2 months after ingestion and missed on endoscopy initially. Further imaging revealed that the fish bone was partially embedded in the pancreatic head surrounded with abscesses and was smoothly removed through laparoscopy.

## 1. Introduction

The ingestion of foreign bodies is a common situation in clinical practice, and most foreign bodies pass through the alimentary canal without any complications. The size, shape, and material of the foreign body determine whether the foreign body will pass through the body without incident or not. Materials with sharp pointed ends, such as fish or chicken bones and needles, are commonly ingested foreign bodies that can cause impaction and possible perforation [1]. In many cases, foreign bodies are found in the esophagus and can be readily removed with flexible endoscopes [2].

Gastrointestinal (GI) tract perforation occurs in approximately 1% of all cases of ingested foreign bodies, and the most common sites are the ileocecal and rectosigmoid regions [3,4]. Only a few cases of abscess formation in affected structures due to penetration by long foreign bodies have been reported. However, when foreign bodies migrate into the deeper layer of an affected organ where they may be difficult to remove endoscopically, surgery is typically required. Herein, we present a rare case of pancreatic abscess secondary to gastric wall perforation by a long fish bone with extraluminal migration; the removal of the fish bone and treatment of the abscess were completed successfully through laparoscopy.

## 2. Case Presentation

**Figure 1:**
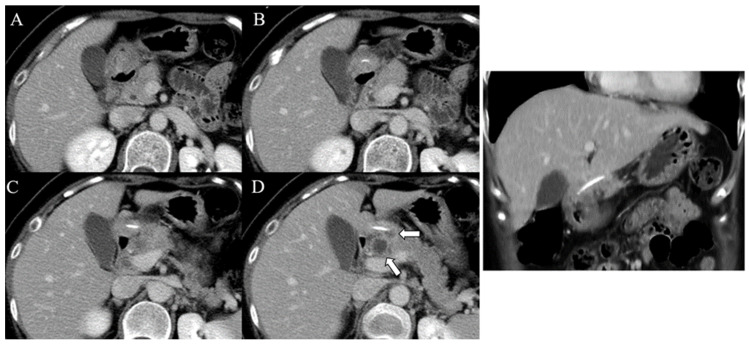
Contrast-enhanced CT imaging with serial sections in the axial (**A**–**D**) and coronal planes displaying a linear and hyperdense foreign body penetrating through the posterior wall of the gastric antrum into the pancreatic head. The white arrows indicate abscess formation in the pancreatic head and near the ingested foreign body.

**Figure 2:**
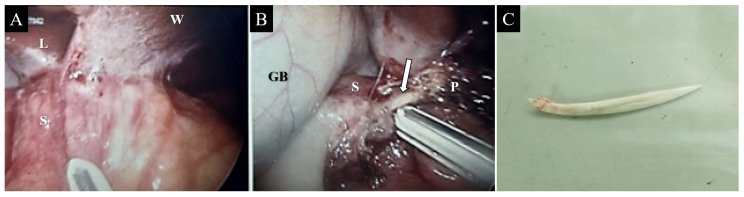
Laparoscopic view displaying severe adhesion between the distal stomach and liver (**A**). A linear and hard foreign body (arrow) was observed between the gastric antrum and the pancreatic head (**B**). The removed foreign body measured 4 cm in length and resembled a fish bone (**C**). Note—L = liver; S = stomach; W = abdominal wall; GB = gallbladder; P = pancreas.

A 49-year-old woman without any underlying conditions presented to our emergency department with worsening dull epigastric pain for over 2 months, general soreness, and fever for 2 days. Physical examination revealed fever up to 39.1 °C with tenderness over the epigastric area; no other complications were reported. Laboratory data indicated a white blood cell count of 8400/µL (neutrophils 88.7%) and a mildly elevated level of C-reactive protein (3.6 mg/dL). The liver function test results and pancreatic enzyme levels were within normal limits. Furthermore, abdominal sonography revealed a small hypoechoic lesion in the hilar area of the liver, and esophagogastroduodenoscopy barely revealed superficial gastritis without mucosal protrusion or ulcer suspiciously indicating the site of foreign body penetration or other etiology. Further investigation was performed through abdominal computed tomography (CT) that revealed a 2.7 cm radiopaque foreign body located between the prepyloric region of the stomach and the pancreatic head and a multiloculated abscess in the pancreatic head and near the aforementioned foreign body (Figure 1). The patient recalled swallowing a piece of fish bone accidentally 2 months prior. Gastric perforation with pancreatic abscess secondary to the fish bone penetration injury was suspected. Considering the length of the fish bone, extraluminal migration, and concurrent multiloculated pancreatic abscess, she was admitted to the hospital and received laparoscopic surgery for removal of the fish bone. The intraoperative findings were a 4 cm long fish bone penetrating through the posterior wall of the gastric antrum into the pancreatic head (Figure 2B,C) and adjoining the pancreatic abscess, and a severe adhesion between the distal stomach and liver, which was identified (Figure 2A) and removed through adhesiolysis. Antibiotic treatment was administered for postoperative symptomatic and supportive treatment. The patient recovered well and was discharged on the seventh postoperative day. No complications were noted in a subsequent health checkup 1 year after the operation.

## 3. Discussion

Penetration injury with further abscess formation caused by ingested fish bone is rarely reported and has been identified mostly in the liver. To date, fewer than 10 cases with concurrent pancreatic abscess have been reported in the English-language literature. The cause might be related to a long, sharp-pointed, and hard fish bone penetrating through the gastric wall during peristalsis with rapid contraction into the adjacent structures, such as the left hepatic lobe near the stomach. In most clinical presentations, the nonspecific features of acute abdominal pain represent a challenging scenario [5]. Furthermore, the lack of a precise history of fish bone ingestion often leads to a diagnostic dilemma and increases morbidity due to the delayed diagnosis of complications and untimely treatment [6]. Imaging studies are highly effective in diagnosing this condition.

Plain film radiography is a commonly used initial screening technique for detecting ingested fish bone; however, it is unreliable because of the variable degrees of radiopacity, depending on the fish species [6]. Ultrasound investigation techniques are also effective for diagnosing abscesses and linear hyperechoic structures resembling fish bones; however, these techniques have a high degree of operator dependence and are subject to interpretive errors. Furthermore, endoscopy helps to concurrently detect and remove objects, but inaccuracies may result in the missing of the embedded fish bone. CT is likely the most effective modality for investigating the cause of unexplained abdominal pain, the exact position of the ingested fish bone, which might not be visible on the endoscope, and the complications associated with its ingestion. CT can reveal a linear hyperdense lesion within the lumen or wall of the GI tract, indicating a fish bone and differentiating it from calcified vessel walls or granuloma [7]. Therefore, in our case, we obtained an accurate preoperative diagnosis based on the history of ingesting a fish bone and corresponding CT findings.

The proper therapeutic methods can be selected according to the size of the abscess cavity and the length, location, and number of fish bones. Since fish bone migrates to the pancreas where endoscopic removal might fail, surgical treatment, including laparoscopic or open approach, is a quite effective management strategy [8]. The laparoscopic surgery is adopted preferably due to its advantages of a small wound, less postoperative pain, lower incidence of wound infection, and rapid postoperative rehabilitation.

Table 1 displays the cases of pancreatic injury due to an ingested fish bone found in a PubMed search (Table 1). In all reported cases, the patient diagnosed with pancreatic abscess presented with symptoms for at least 1 week. The average length of the ingested fish bones was 3.1 cm; the bones were safely removed through laparoscopic surgery despite a large part of the bone being embedded in the pancreas. Compared with cases that underwent open surgery (OS), patients who underwent laparoscopic surgery (LS) had significantly shorter postoperative day discharge (9.4 ± 2.1 days in OS and 5.3 ± 1.5 in LS). In our case, the length of the fish bone was 4 cm, which is the longest among the others previously reported to be the cause of pancreatic abscesses. Also, the only case with an abscess removed laparoscopically had neither prolongation of hospitalization days nor complications. Therefore, laparoscopic approach should be considered as a priority especially in this series.

In conclusion, complications due to fish bone ingestion are rare and typically pre-sent uncharacteristically. To date, there are still no established standard diagnostic tools or guidelines for treatment. Contrast-enhanced CT should be performed to evaluate unexplained and persistent abdominal discomfort and obtain crucial information regarding the relationship of an endoscopically undetectable foreign body with surrounding organs before selecting a therapeutic approach. Laparoscopic surgery is a safe and quick procedure for removing a long penetrating fish bone embedded in a solid organ and surrounding a multiloculated abscess simultaneously, as in our case [6]; this technique is also associated with shorter postoperative length of stay at the hospital and few complications.

## Figures and Tables

**Table 1:** Reported cases of pancreatic injury due to penetration by ingested fish bone.

Author	Patient Details	Symptoms/Duration	Fish Bone Size (cm)	Site of Perforation	Abscess	Management	POD Discharge (Day)
Goh B.K. et al., 2004 [7]	60 Y/O, F	Epigastric discomfort/ 2 weeks	3	Stomach	+	Laparotomy	11
Goh B.K. et al., 2005 [1]	32 Y/O, M	Fever with chills/ 5 days	3	Duodenum	+	Laparotomy	9
Wang W.L. et al., 2008 [9]	68 Y/O, M	Dull epigastric pain/ 4 weeks	2.3	Stomach	+	Laparotomy	8
Symeonidis D. et al., 2012 [10]	57 Y/O, F	Mid-epigastric pain, nausea and vomiting/ 1 day	N/A	Duodenum	−	Laparotomy with duodenotomy	7
Huang Y.H. et al., 2013 [4]	53 Y/O, F	Dull abdominal pain/ 3 months	3.2	Stomach	+	Laparotomy	12
Mima K. et al., 2018 [11]	80 Y/O, M	Epigastric pain/1 day	2.5	Stomach	−	Laparotomy	7
Attila T. et al., 2019 [12]	76 Y/O, M	Epigastric pain/2 days	3.5	Duodenum	−	Laparotomy	N/A
Wang Y. et al., 2021 [6]	67 Y/O, M	Mild epigastric pain/ 3 months	3.2	Stomach	−	Laparoscopic surgery	5
Mulita, F. et al., 2021 [13]	50 Y/O, F	Epigastric pain/N/A	3	Stomach	−	Laparoscopic surgery	4
Our case	49 Y/O, F	General soreness, fever, dull epigastric pain/ 2 months	4	Stomach	+	Laparoscopic surgery	7

Note—M = male; F = female; + = positive; − = negative; POD = postoperative day.

## Data Availability

All data are available within the article.
